# Disclosure is not destigmatization: rethinking AI and mental health stigma

**DOI:** 10.3389/fpubh.2026.1881272

**Published:** 2026-06-29

**Authors:** Weina Chen, Zhirou Zeng, Weizhi Liu, Wenjie Yan

**Affiliations:** 1 Lab for Post-Traumatic Stress Disorder, Faculty of Psychology and Mental Health, Naval Medical University, Shanghai, China; 2 Department of Psychology, University of Toronto Scarborough, Toronto, ON, Canada

**Keywords:** artificial intelligence, chatbot, digital mental health, disclosure, stigma

## Introduction

The rapid expansion of generative artificial intelligence (AI) has accelerated the integration of conversational agents into mental health support systems (1–6). Large language model (LLM)-based tools are increasingly used for emotional support, psychoeducation, symptom discussion, and help-seeking guidance (2, 3, 5, 7–9). Existing evidence suggests that some users perceive AI systems as less judgmental and emotionally safer environments for discussing stigmatized or psychologically sensitive concerns (9–11).

This trend has contributed to growing optimism regarding the anti-stigma potential of AI-assisted mental health support. In particular, recent studies suggest that individuals may feel more comfortable disclosing distress, symptoms, or emotionally sensitive experiences to AI systems than in some interpersonal contexts, including clinical or peer-based interactions (9, 12–15). Consequently, greater willingness to engage with AI tools has often been interpreted as evidence that AI may alleviate stigma-related barriers and facilitate psychological help-seeking (11, 13, 15, 16). Such interpretations, however, depend on how stigma is conceptualized and measured. Mental health stigma is a multidimensional construct encompassing public stigma, self-stigma, structural stigma, and anticipated stigma. Among these forms, anticipated stigma, defined as expectations of being judged, rejected, or devalued by others, is particularly relevant to disclosure decisions and help-seeking behavior (17, 18). Here, disclosure refers to the voluntary communication of mental health concerns to another person or system, including AI-mediated interactions (19).

Importantly, increased disclosure does not necessarily imply reduced stigma. Individuals may become more willing to discuss mental health concerns with AI systems while continuing to anticipate judgment, conceal symptoms, or avoid disclosure in human social contexts. Existing research suggests that AI systems may reduce some interpersonal barriers to disclosure by providing environments perceived as less judgmental, less socially evaluative, and more comfortable for discussing sensitive concerns (10–16). The distinction between increased disclosure and stigma reduction is consistent with both disclosure theory (20–22) and the Disclosure Process Model [DPM; (19)]. The DPM conceptualizes disclosure as an ongoing interpersonal process rather than a single event, emphasizing that disclosure outcomes depend on contextual and interpersonal factors, including disclosure goals, characteristics of the disclosure target, and social responses following disclosure. From this perspective, AI-assisted disclosure may represent changes in the conditions under which disclosure occurs rather than reductions in underlying stigmatizing attitudes or expectations. Consequently, evidence that AI facilitates disclosure should not be automatically interpreted as evidence that AI reduces mental health stigma.

A person may feel comfortable disclosing depressive symptoms to an AI chatbot while still avoiding disclosure to clinicians, family members, employers, or peers. This possibility is consistent with broader disclosure research showing that disclosure decisions and outcomes vary substantially across social contexts and intended audiences (19, 23). Prior research has shown that anonymous or less identifiable online environments can facilitate self-disclosure by reducing social risks and concerns about negative evaluation (24–26). Consistent with this, individuals frequently use online platforms to discuss stigmatized experiences, mental health concerns, and other sensitive issues that may be difficult to disclose in offline settings (27, 28). Taken together, these findings may be more consistent with the interpretation that increased disclosure to AI systems reflects the availability of a lower-risk disclosure environment rather than genuine reductions in mental health stigma.

## The strongest evidence for stigma reduction comes from purpose-built interventions

A relatively small but coherent group of studies provides evidence that AI systems can reduce certain forms of mental illness stigma when they are explicitly designed as anti-stigma interventions (29–32). These interventions typically incorporate mechanisms such as guided social contact, empathy induction, perspective taking, emotional engagement, or educational framing rather than simply providing a private AI conversation partner.

Across chatbot-based social contact interventions, vulnerable social bots, and embodied conversational agents, studies have reported reductions in blame, fear-related stigma, and desired social distance, alongside increases in helping intentions and other supportive attitudes toward individuals with mental illness (29–32). However, these findings also suggest that the active mechanism is not AI exposure itself. Rather, stigma reduction appears most evident when AI systems are intentionally designed to deliver contact-based, educational, or emotionally guided interventions. Such approaches differ fundamentally from ordinary AI-mediated emotional disclosure, and their effectiveness should not be generalized into the broader claim that AI inherently reduces stigma.

## Much of the broader enthusiasm may actually reflect reduced exposure

Outside these purpose-built interventions, much of the AI mental health literature examines a different outcome: increased willingness to disclose distress to AI systems. For example, Lucas et al. (15) found that service members reported more mental health symptoms to a virtual human interviewer than during standard post-deployment mental health assessments. This finding suggests that AI-mediated interactions may facilitate disclosure, although the study did not directly assess changes in stigma-related attitudes or beliefs. More recent studies suggest that AI systems become especially attractive in contexts characterized by stigma-related concerns, including shame, anticipated judgment, embarrassment, and fear of social evaluation (11, 13, 16, 33–35). Individuals who fear negative evaluation from others may perceive AI systems as emotionally safer disclosure environments because such systems are often experienced as less judgmental, less socially evaluative, and more accepting than many interpersonal interactions (1, 9, 12, 14, 15).

Collectively, these findings suggest that AI systems may reduce the interpersonal costs of disclosure by providing emotionally safer interaction environments.

However, reduced disclosure burden should not automatically be equated with reduced stigma. Individuals may become more willing to disclose distress to AI systems while continuing to conceal these experiences from clinicians, family members, or peers. In this sense, AI systems may function less as direct anti-stigma tools and more as low-risk alternatives to interpersonal disclosure.

## Why disclosure alone should not be treated as evidence of destigmatization

Several conceptual and methodological considerations suggest that disclosure alone should not be treated as a direct proxy for stigma reduction. First, the outcomes measured in many AI mental health studies differ substantially from traditional stigma outcomes. Existing studies frequently assess willingness to disclose, emotional openness, acceptability, or intention to engage with AI systems (1, 3, 11, 12, 16, 33–37). While clinically relevant, these outcomes are not equivalent to reductions in self-stigma, public stigma, discriminatory attitudes, or desired social distance.

Second, much of the current evidence remains correlational or cross-sectional. Although shame-related barriers, anticipated stigma and concerns about social evaluation are associated with greater willingness to use AI support tools (11, 13, 34–36), such findings cannot determine whether AI interactions actually reduce stigma over time. Third, the same characteristics that facilitate AI-based disclosure may leave the social roots of stigma largely unchanged. Although AI systems provide private and low-risk interaction environments (2, 7, 9–12, 15, 37), these features do not necessarily alter how mental illness is perceived within families, workplaces, schools, peer groups, or healthcare systems (17, 18, 38–40).

For this reason, AI systems may currently function more accurately as stigma buffers than as direct stigma-reduction tools, reducing exposure to stigmatizing interactions without necessarily changing stigma itself. Such systems may create lower-friction pathways into emotional disclosure, screening, or referral while leaving broader social attitudes toward mental illness relatively intact.

## AI systems may also reproduce stigma

Strong claims regarding the anti-stigma potential of AI must also account for evidence that current AI systems may themselves reproduce stigmatizing content. Studies evaluating large language models in mental health contexts have reported stigmatizing responses, problematic therapeutic interactions, and negative judgments toward individuals with mental illness, including borderline personality disorder and substance use disorders (8, 41–44). These findings suggest that stigma may emerge not only from users' concerns about disclosure but also from the content generated by AI systems themselves (8, 45).

Ease of disclosure and quality of representation are therefore distinct properties. A chatbot may lower barriers to disclosure while simultaneously reinforcing harmful assumptions, biased language, or stigmatizing narratives. Consequently, increased user comfort should not be interpreted as evidence that AI systems promote psychologically safer or less stigmatizing understandings of mental illness.

## Discussion

The current literature does not yet fully support the broad claim that AI-assisted mental health disclosure reduces stigma itself. A more conceptually precise interpretation is that AI systems may play two distinct roles.

First, carefully designed AI interventions can reduce stigma through structured social contact, empathy induction, perspective taking, and educational framing. Second, and more commonly, AI systems appear to reduce the interpersonal cost of disclosure by providing privacy, anonymity, reduced social evaluation, and emotionally safer interaction environments. The first process reflects genuine anti-stigma intervention. The second may be better understood as stigma buffering or stigma avoidance, in which AI systems reduce the interpersonal burden of disclosure without necessarily changing underlying social attitudes toward mental illness. We suggest that distinguishing between stigma reduction and stigma buffering may provide a more precise framework for evaluating the role of AI in mental health support. While stigma-reduction interventions aim to change stigmatizing attitudes, beliefs, or social structures, stigma-buffering tools primarily reduce the interpersonal costs associated with disclosure and help-seeking. Recognizing this distinction may help researchers more accurately interpret the benefits and limitations of AI-assisted mental health support.

Future mental health interventions may benefit from integrating traditional stigma-reduction approaches, such as contact-based interventions, psycho-education, and perspective-taking programs, with AI-based support tools that reduce disclosure barriers and facilitate initial help-seeking. Such an approach may help combine the strengths of stigma-reduction and stigma-buffering strategies within a single intervention framework. From a research perspective, future studies should move beyond disclosure willingness alone and more directly examine whether AI-assisted interactions reduce fear of human judgment, improve real-world help-seeking behavior, and produce durable changes in interpersonal and institutional attitudes toward mental illness.

As AI systems become increasingly integrated into mental health care, distinguishing between stigma reduction and reduced interpersonal exposure may become essential for evaluating AI-assisted support and defining meaningful anti-stigma progress in the digital era.
